# Enhanced electrocaloric analysis and energy-storage performance of lanthanum modified lead titanate ceramics for potential solid-state refrigeration applications

**DOI:** 10.1038/s41598-017-18810-z

**Published:** 2018-01-10

**Authors:** Tian-Fu Zhang, Xian-Xiong Huang, Xin-Gui Tang, Yan-Ping Jiang, Qiu-Xiang Liu, Biao Lu, Sheng-Guo Lu

**Affiliations:** 1 School of Physics & Optoelectric Engineering, Guangdong University of Technology, Guangzhou Higher Education Mega Center, Guangzhou, 510006 People’s Republic of China; 2 School of Materials & Energy, Guangdong Province Key Lab Function Soft Matter, Guangdong University of Technology, Guangzhou Higher Education Mega Center, Guangzhou, 510006 People’s Republic of China

## Abstract

The unique properties and great variety of relaxer ferroelectrics make them highly attractive in energy-storage and solid-state refrigeration technologies. In this work, lanthanum modified lead titanate ceramics are prepared and studied. The giant electrocaloric effect in lanthanum modified lead titanate ceramics is revealed for the first time. Large refrigeration efficiency (27.4) and high adiabatic temperature change (1.67 K) are achieved by indirect analysis. Direct measurements of electrocaloric effect show that reversible adiabatic temperature change is also about 1.67 K, which exceeds many electrocaloric effect values in current direct measured electrocaloric studies. Both theoretical calculated and direct measured electrocaloric effects are in good agreements in high temperatures. Temperature and electric field related energy storage properties are also analyzed, maximum energy-storage density and energy-storage efficiency are about 0.31 J/cm^3^ and 91.2%, respectively.

## Introduction

Since the discovery of ferroelectrics, ferroelectric materials have been exploited in many applications, such as: piezoelectric energy harvesting, optical electronic devices, and *etc* ^1–3^. The unique properties and great variety of relaxer ferroelectrics also make them highly attractive for future solid-state refrigeration technologies. During the past decades, intensive research efforts have been conducted to develop solid-state cooling technologies^3,4^. The adiabatic temperature change (Δ*T*) and isothermal entropy change (Δ*S*) of polar materials are figure of merits of electrocaloric effect (*ECE*) during application and removal of electric field, which is environment friendly. *ECE* provides a highly efficient approach to achieve solid-state cooling instead of the existing vapour-compression refrigeration^5–9^. Recently, ferroelectrics for future solid-state refrigeration technologies become very hot^10–18^. In order to gain higher Δ*T*, many scholars pay attentions to thin films due to their large breakdown field^19–21^. It is a well-known fact that thin films have advantages in small solid state cooling devices, but bulk materials play an important role on larger scale devices, such as: refrigeration^22,23^. As a result, *ECE* of bulk materials are also desired, we should pay more attentions to *ECE* of bulk materials. Bulk materials including multilayer capacitors, ceramics and single crystals have been reported a lot, such as: 0.9Pb(Mg_1/3_Nb_2/3_)O_3_–0.1PbTiO_3_ multilayer capacitors^16^, 0.9PMN-0.1PT single crystal^14^, Ba_1−*x*_Sr_*x*_TiO_3_ ceramics^12^. Compared to multilayer capacitors and single crystals, ceramics have the advantages of low-cost and easier fabrications.

In recent years, lead titanate (PT) based ceramics become one of the most studied and used ferroelectric materials in both scientific and industrial communities due to its high Curie temperature (*T*_c_) and low dielectric constant^24,25^, which make PT based ceramic to be a valuable research object^26–30^. In this work, lanthanum modified lead titanate ceramics (Pb_1−*x*_La_*x*_)Ti_1−*x*/4_O_3_ (PLT100*x*, *x* = 0.20, 0.24, 0.28, and 0.32, abbreviated as PLT20, PLT24, PLT28 and PLT32 respectively) ceramics are prepared and studied. Energy-storage and *ECE* of PLT ceramics are revealed for the first time. In this work, frequencies and temperatures dependent dielectric permittivity *ɛ*_*γ*_ and loss *tanδ* are also investigated to study the relaxer phase transitions and defects related relaxations. Ferroelectric based energy-storage properties are also analyzed. Energy-storage density in this work researches about 0.31 J/cm^3^, high energy-storage efficiency (91.18%) is also obtained. Large *ECE* in PLT ceramics is achieved for the first time, maximum value of Δ*T* is about 1.67 K, and giant refrigeration efficiency is up to 27.4. Additional direct measured electrocaloric effects are analyzed, giant temperature change (1.67 K) is achieved, which indicates that PLT ceramics may be used in future solid-state refrigeration applications.

## Experimental

PLT ceramics were synthesized by a conventional high temperature solid-state fabrication method. Reagent-grade Pb_3_O_4_, La_2_O_3_ and TiO_2_ powders were weighted according to their stoichiometric composition. Then powders were first mixed and calcined at 850 °C for 5 h. The calcined powders were then mixed with alcohol milling for 24 h and dried. After that, powders were mixed thoroughly with a polyvinyl alcohol (PVA) binder solution and pressed into discs of 10 mm in diameter and 1 mm in thickness uniaxially. These discs were sintered at 1300 °C for 2 h in air. Silver paste was applied on both sides of discs and fired at 650 °C as electrodes for electrical properties measurements. High temperature dielectric behaviours were measured by Agilent E4980A (measure conditions: 0.5–1000 kHz, 25–600 °C). Low temperature permittivity *ɛ*_*γ*_ and dielectric loss *tanδ* of PLT samples were measured using an HP4194A LCR (measured conditions: 0.1–100 kHz, −193–165 °C). Complex impedance plots were conducted by Agilent E4980A (0.02–2000 kHz). Ferroelectric hysteresis loops were obtained by a computer-controlled virtual-ground circuit with Precision Premier II Ferroelectric Tester (Radiant Technologies, Inc., Albuquerque, New Mexico, USA). The direct measurements of *ECE* were conducted by a customized system: for the direct measurement, *ECE* change of temperature was monitored by a small thermistor attached to the upper gold electrode of ceramic. In order to reduce the heat exchange with environment, a thermistor and an electric field controlled by a computer were employed to detect the temperature change caused by *ECE* as the application or withdrawing of an electric field. Also, a high voltage generator controlled by an arbitrary signal generator is used to generator the electric field step signal, which is then applied to the sample. The voltage should be maintained for a few seconds to get into thermal equilibrium with the surrounding. Then the voltage was released immediately. The typical thermal response times along the sample thickness direction is a few milliseconds. Within such a short period, a very fast equilibration of the temperature throughout the whole sample, including the electrodes, attached thermistor and wires, took place, but then the equilibrated sample exchanges the heat on a much longer time scale to the surrounding bath.

## Results and Discussion

**Figure 1:**
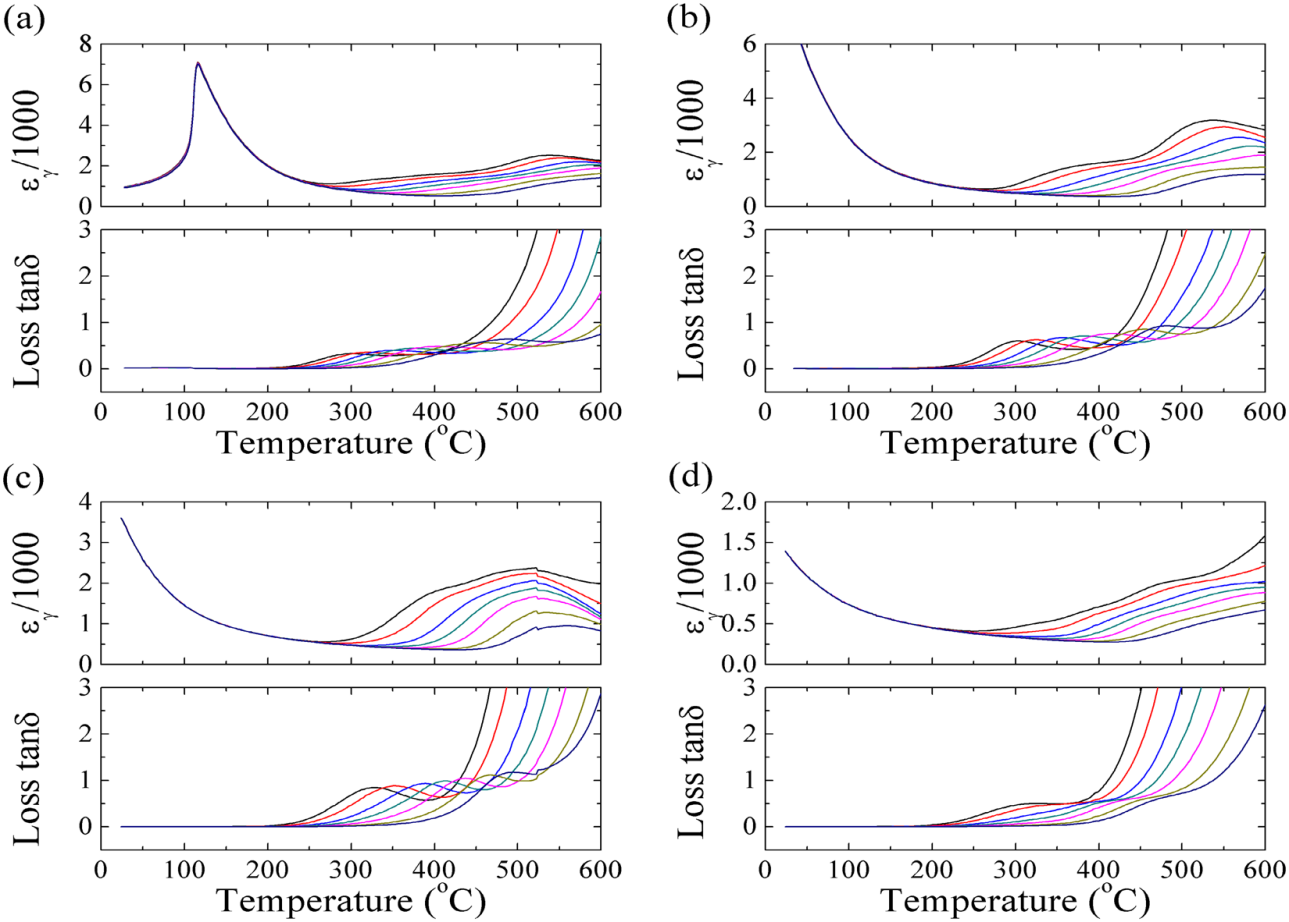
Dielectric permittivity *ε*_*γ*_ and dielectric loss *tanδ* as a function of temperatures for (**a**) PLT20, (**b**) PLT24, (**c**) PLT28 and (**d**) PLT32 ceramics with various measured frequencies (1, 2, 5, 10, 20, 50 and 100 kHz).

**Figure 2:**
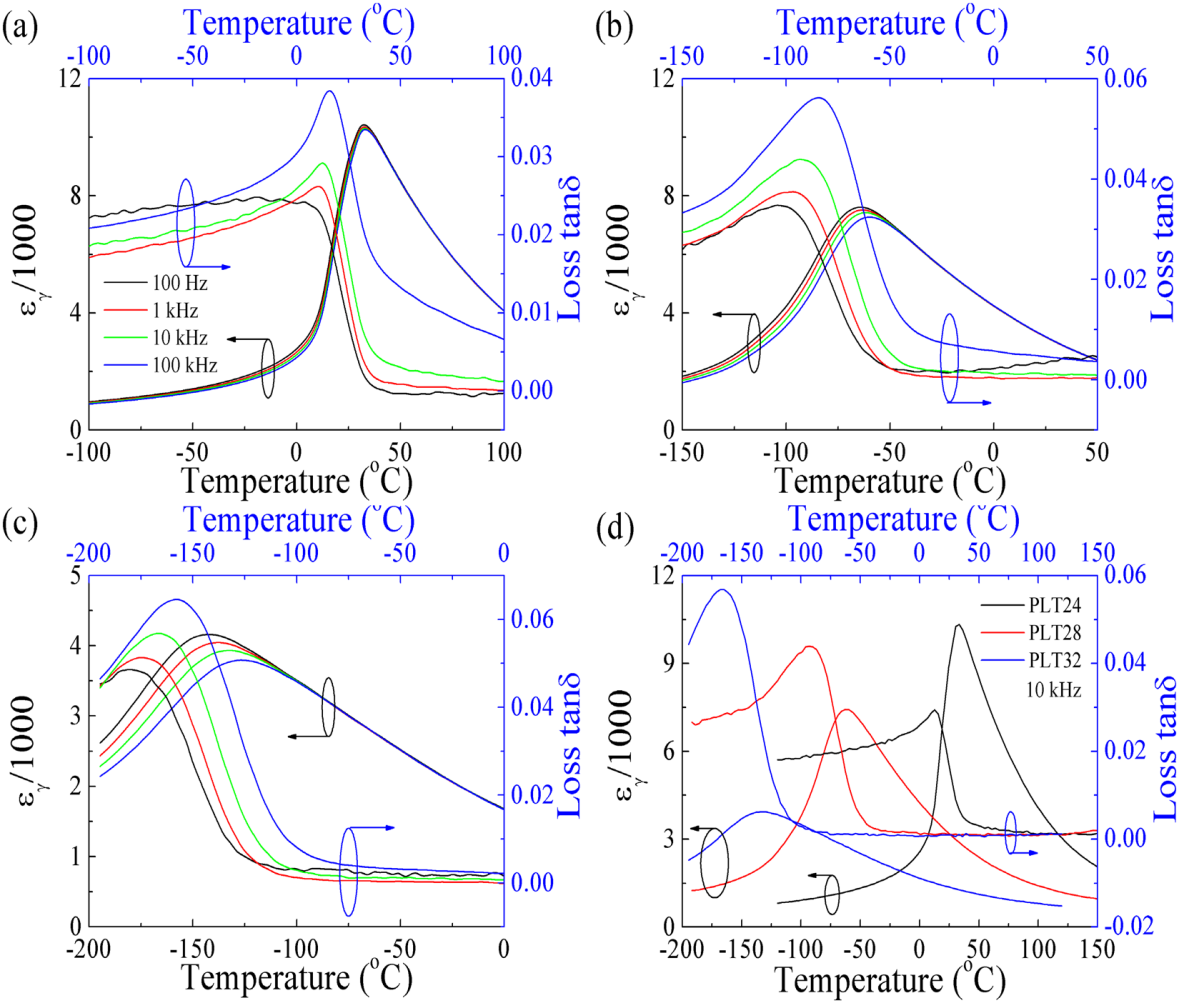
Dielectric permittivity *ε*_*γ*_ and dielectric loss *tan*δ as a function of temperatures for (**a**) PLT24, (**b**) PLT28 and (**c**) PLT32 ceramics, (**d**) three samples at 10 kHz.

Temperatures dependent dielectric permittivity *ɛ*_*γ*_ and loss *tanδ* for PLT samples are shown in Fig. 1 (Room temperature to 600 °C) and Fig. 2 (Lower temperatures: −193–165 °C). From Fig. 2, Temperature dependent *ɛ*_*γ*_ depicts typical relaxer behaviours with a strong dispersion of *ɛ*_*γ*_ peaks, especially for PLT28 and PLT32 ceramics, *T*_*m*_ (temperature of maximum *ɛ*_*γ*_) shift to higher temperatures and maximum *ɛ*_*γ*_ decrease with increasing frequencies. On the other hand, loss *tanδ* also exhibits broad peaks clearly, with increasing frequencies, maximum loss *tanδ* increase as well. Similar results were also reported^31,32^. This phenomenon signifies relaxer behaviours^33^.

**Figure 3:**
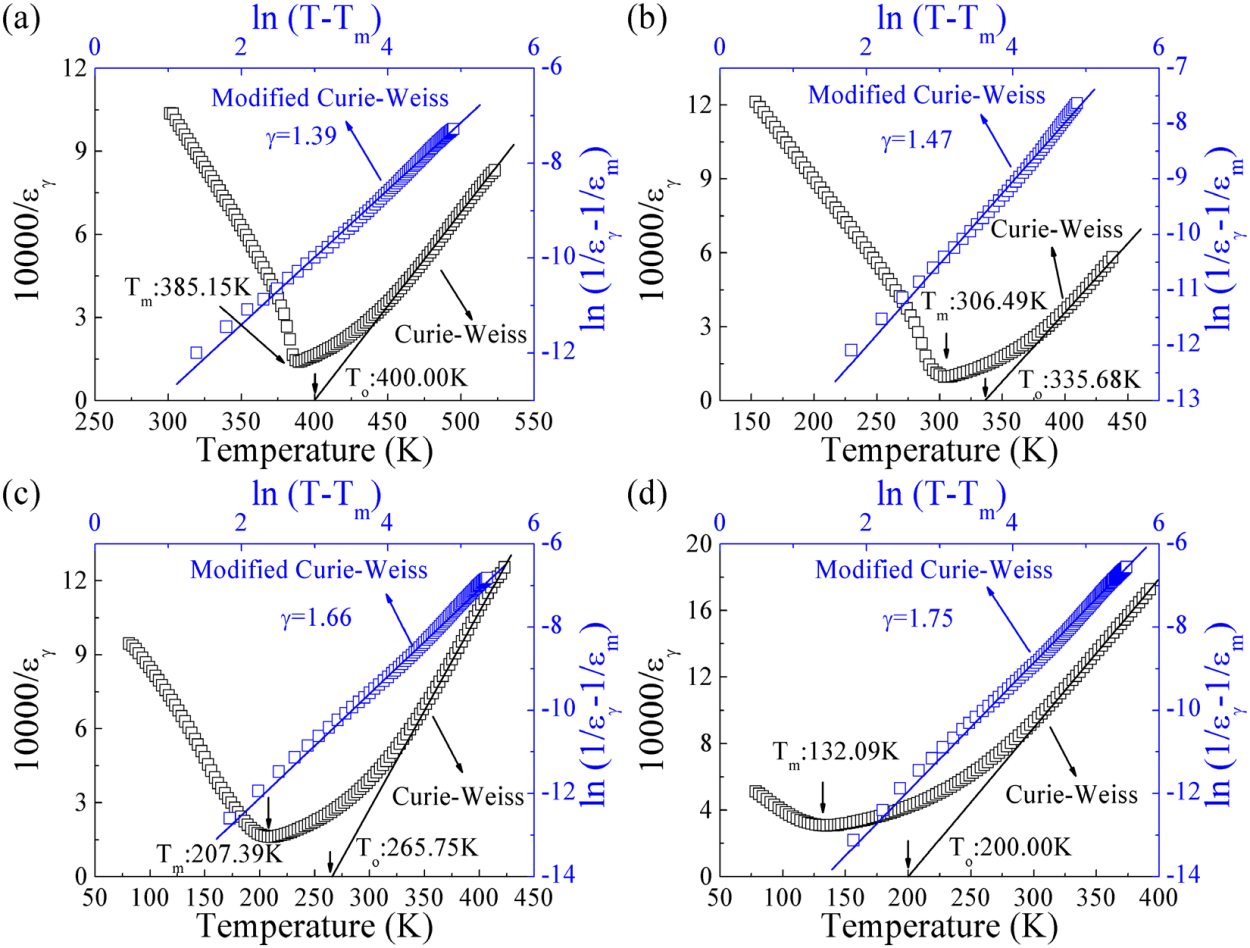
The inverse of dielectric permittivity (10000/*ε*_*γ*_) as a function of temperature at 10 kHz (The black solid lines are used to fit the Curie-Weiss law), and the plot of ln(1/*ε*_*γ*_ − 1/*ε*_*m*_) as a function of ln(*T* *−* *T*_*m*_) for PLT ceramics measured at 10 kHz (The blue solid lines are a fit of modified Curie-Weiss relationship) respectively for (**a**) PLT20, (**b**) PLT24, (**c**) PLT28 and (**d**) PLT32 ceramics.

Generally speaking, the maximum value of *ɛ*_*γ*_, at the Curie point *T*_*c*_ of an ideal ferroelectric crystal can be described by the Curie-Weiss law^34^:

1 $$1/{\varepsilon }_{\gamma }=(T-{T}_{o})/C,(T > {T}_{C})$$

where *C* and *T*_*o*_ are Curie-Weiss constant and Curie-Weiss temperature, respectively. For a first-order phase transition, *T*_*C*_ is greater than *T*_*o*_, whereas for second-order phase transitions, *T*_*C*_ equals *T*_*o*_^34^. In this work, *ɛ*_*γ*_ of PLT ceramics are analyzed by the Curie-Weiss law, plots of temperatures versus inverse *ɛ*_*γ*_ (at 10 kHz) are shown in Fig. 3. *T*_*m*_ and *T*_*o*_ are 385.15 K and 400.00 K, 306.49 and 335.68 K, 207.39 K and 265.75 K, 132.09 and 200.00 K respectively for PLT20, PLT24, PLT28, and PLT32 ceramics. Clearly, both *T*_*m*_ and *T*_*o*_ decrease sharply with increasing La concentrations.

It is well known that dielectric behaviours of relaxer ferroelectrics exhibit to deviate from typical Curie-Weiss behaviour, it can be described by a modified Curie-Weiss relationship^35^:

2 $$1/{\varepsilon }_{\gamma }-1/{\varepsilon }_{m}={(T-{T}_{m})}^{\gamma }/{C}_{1},(1\le \gamma \le 2)$$

where *C*_1_ and *γ* are assumed to be constant, and *ɛ*_*m*_ is the maximum permittivity. Parameter *γ* shows clear information on the character of phase transitions^36–38^. Figure 3 shows the plots of ln(1/*ɛ*_*γ*_ − 1/*ɛ*_*m*_) versus ln(*T* − *T*_*m*_) with (at 10 kHz). After fitting the experimental data to the modified Curie-Weiss relationship, we obtain the value of parameter *γ* = 1.39, 1.47, 1.66, 1.75, respectively for PLT20, PLT24, PLT28 and PLT32 ceramics. Fitting values of *γ* also support the evidence of relaxer nature.

**Figure 4:**
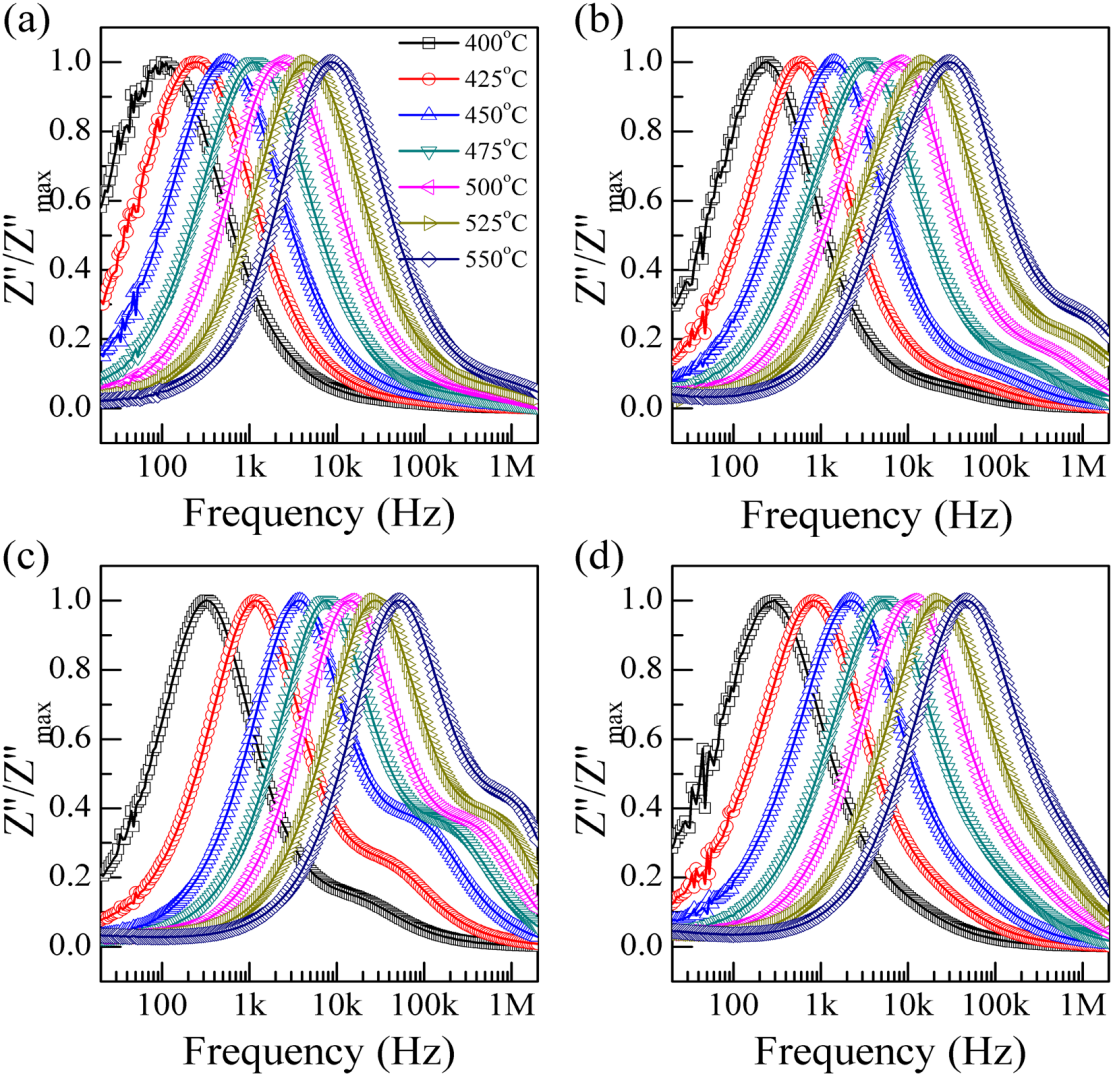
Normalized imaginary parts *Z*″*/Z*″_*max*_ of impedance as a function of frequencies for (**a**) PLT20, (**b**) PLT24, (**c**) PLT28 and (**d**) PLT32 ceramics.

**Figure 5:**
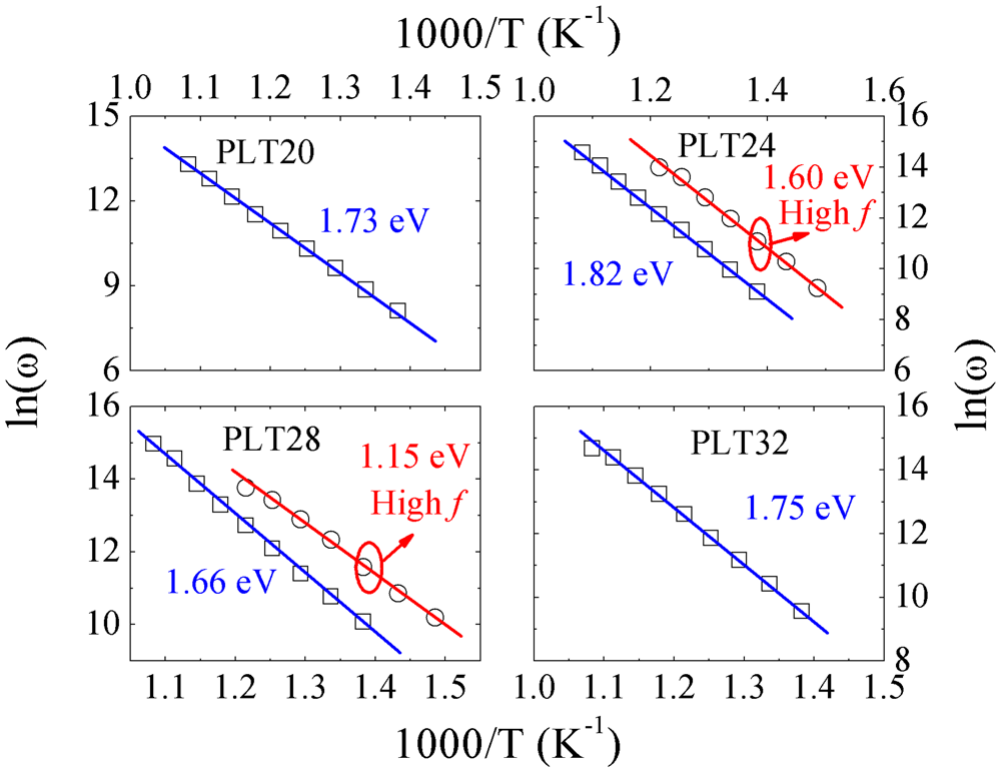
ln*ω* versus 1000/T curves for PLT ceramics, straight lines are used to fit the Arrhenius law.

From Fig. 1, it is found that abnormal dielectric peaks in permittivity and loss are observed (higher temperature region), similar behaviours are also reported in other perovskites (10–10^7^ Hz, 400–800 °C), which are called dielectric relaxation^38–41^. In order to give a clear knowledge of high temperature dielectric relaxations, impedance technology is selected as an efficient technique, which has been intensive used in electrical properties of electro-ceramic materials^42^. The variation of normalized imaginary parts of impedance (*Z*″/*Z*″_*max*_) are shown in Fig. 4. Clearly, values of *Z*″/*Z*″_*max*_ become close gradually in higher frequencies. For PLT24 and PLT28 ceramics, *Z*″/*Z*″_*max*_ can be fitted into 2 separate parts. For a thermally activated relaxation process, relaxation frequency usually follows the Arrhenius law:

3 $$\omega ={\omega }_{o}\cdot exp(-{E}_{a}/{k}_{\beta }T)$$

where *T*, *ω*_*o*_, *E*_*a*_, *k*_*β*_ are the absolute temperature, characteristic frequency, activation energy and Boltzmann constant, respectively. Relaxation parameter *E*_*a*_ is determined by plotting *ln*(*ω*) as a function of the inverse of temperature (1000/*T*) using Arrhenius law (shown in Fig. 5). Two independent activation energies are obtain for PLT24 and PLT28 ceramics, grains (high frequency) and grain boundaries (low frequency) related activation energies are 1.60 eV and 1.82 eV, 1.15 eV and 1.66 eV respectively. Values of grain boundaries related activation energy are higher than those of grains, this indicate that grain boundaries exhibit higher resistance than grains^43^. For PLT20 and PLT32 ceramic, activation energies are about 1.73 and 1.75 eV respectively. For all compositions, values of activation energies are very close to OVs related high temperature dielectric relaxations in perovskite systems, such as: SrTiO_3_^44^, (PbLa)(Zr_0.9_Ti_0.1_)O_3_^45^, and (Pb,Cd,La)TiO_3_ ceramics^42^.

**Figure 6:**
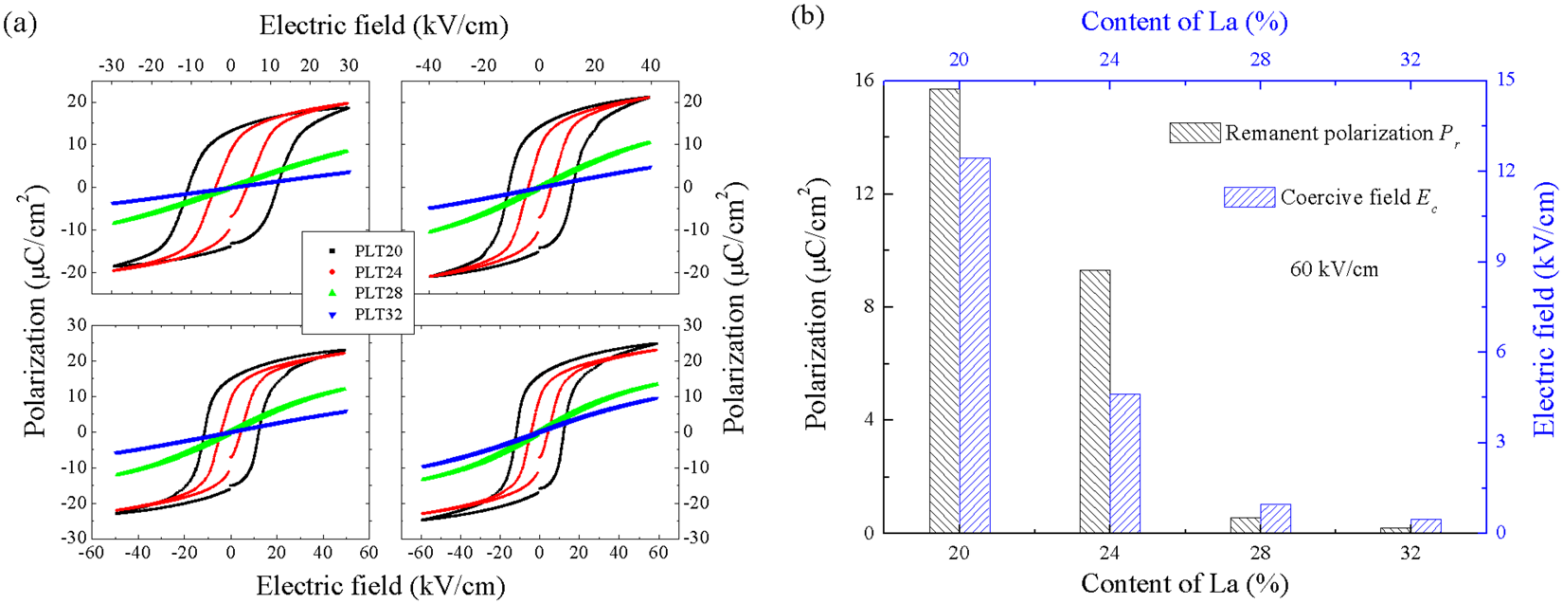
(**a**) *P-E* loops of PLT ceramics measured under various electric fields for PLT20 (black line), PLT24 (red line), PLT28 (blue line), PLT32 (magenta line). (**b**) Comparison of remanent polarization and coercive field of all samples.

Figure 6a shows polarization-electric field (*P-E*) hysteresis loops of PLT ceramics under various electric fields (30–60 kV/cm, ~300 K, 20 Hz). Typical ferroelectric hysteresis loops are observed for PLT20 and PLT24 ceramics, which manifests the ferroelectric phase at room temperatures. For PLT28 and PLT32 ceramics, slim hysteresis loops are achieved indicating the relaxer ferroelectric nature. At room temperature, remnant polarization, and coercive field decrease sharply with increasing La concentrations as shown in Fig. 6b.

As a well-known fact, *P-E* hysteresis loops also reflect energy-storage capacities of dielectric materials. According to the definition of energy-storage density by *P-E* hysteresis loops, energy-storage density, *J*_*reco*_, is defined as^46–49^:

4 $${J}_{reco}=\int EdP$$

**Figure 7:**
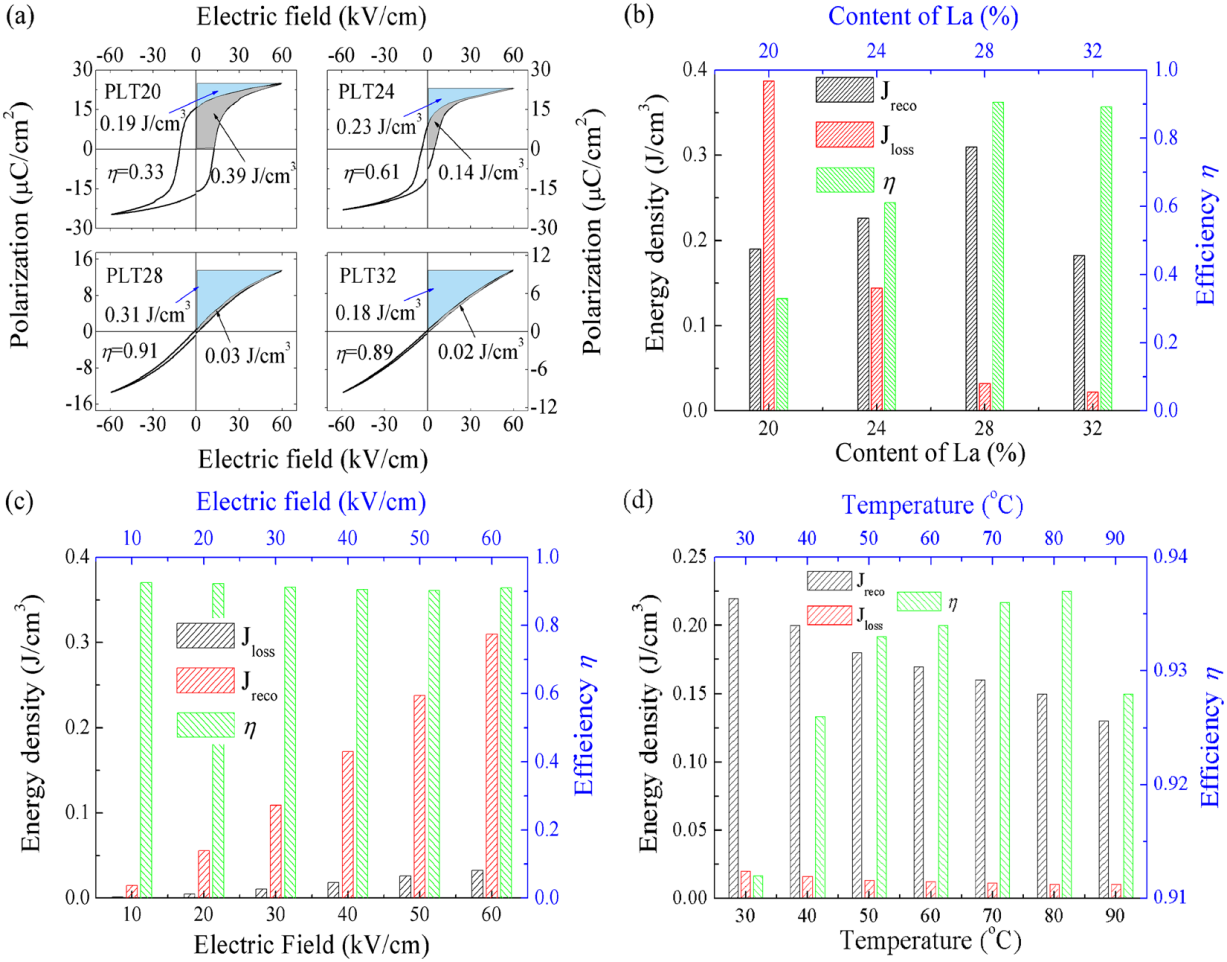
(**a**) Energy storage density calculated from *P-E* hysteresis loops of PLT ceramics, the blue area and the gray area showed the energy-storage density and energy-loss density, respectively. (**b**) Energy-storage properties with as a function of La concentrations. (**c**) Electric fields and (**d**) temperatures influenced energy-storage properties of PLT28 ceramic.

Based on the above formula, *J*_*reco*_ can be obtained by numerical integration of the area between polarization axis and curves of *P-E* loops easily. In this work, energy-storage density *J*_*reco*_ (blue area, shown in Fig. 7a) calculated from *P-E* loops are about 0.19, 0.23, 0.31 and 0.18 J/cm^3^ respectively for PLT20, PLT24, PLT28 and PLT32 ceramic (at 60 kV/cm). From the aspect of practical application, high energy-storage efficiency (*η*) and low energy-loss density (*J*_*loss*_) are also significant. Similar to energy-storage density (*J*_*reco*_), energy-loss density *J*_*loss*_ (the gray area, shown in Fig. 7a) can also be calculated from *P-E* loops. Results revealed that *J*_*loss*_ was about 0.39, 0.14, 0.03 and 0.02 J/cm^3^ for PLT20, PLT24, PLT28 and PLT32 ceramic. Energy-storage efficiency *η* is defined as^50–53^:

5 $$\eta ={J}_{reco}/({J}_{reco}+{J}_{loss})$$

Accordingly, room temperature energy-storage efficiency of PLT ceramics according to the above formula are about 33%, 61%, 91%, and 89% respectively for PLT20, PLT24, PLT28 and PLT32 ceramic. Figure 7b shows the influence of La concentrations on the energy-storage properties. Clearly, PLT28 shows better energy-storage properties.

Due to the higher energy-storage density and efficiency of PLT28, PLT28 ceramics are chosen to study the influence of measured temperatures and electric fields on energy-storage properties (shown in Fig. 7c,d). Clearly, both *J*_*reco*_ and *J*_*loss*_ increase sharply with increasing electric fields, because larger electric field can induce higher polarization, and higher polarization will increase energy-storage density inevitably, but *η* keeps stable under various electric fields (>90%) (See Fig. 7c). With increasing temperatures, both *J*_*reco*_ and *J*_*loss*_ decrease sharply due to the decreasing of polarizations, but *η* exhibits the maximum value at 80 °C.

In this work, our results show lower values of *J*_*reco*_ than (Pb_0.91_La_0.09_)(Zr_0.65_Ti_0.35_)O_3_ relaxer ferroelectric thin films^54^, PbZrO_3_ antiferroelectric thin films^55^, HfO_2_-ZrO_2_ solid solution thin films^56^, and HfZrO_2_ films^53^, those works report very good results, the energy storage densities are rather large. Compared with bulk materials, our results of *J*_*reco*_ are higher than BaTiO_3_-SrTiO_3_ composites^57^, BaSrTiO_3_ ceramics^58^, Sr_0.5_Ba_0.5_Nb_2_O_6_ glass-ceramics^59^, and *etc*. From aspects of applications, the priority among priorities for energy-storage devices are to gain a slim hysteresis loop (large saturated polarization, weak coercive field and small remanent polarization) or double hysteresis loops, this is the research direction to which we should pay more attentions^47,52^.

**Figure 8:**
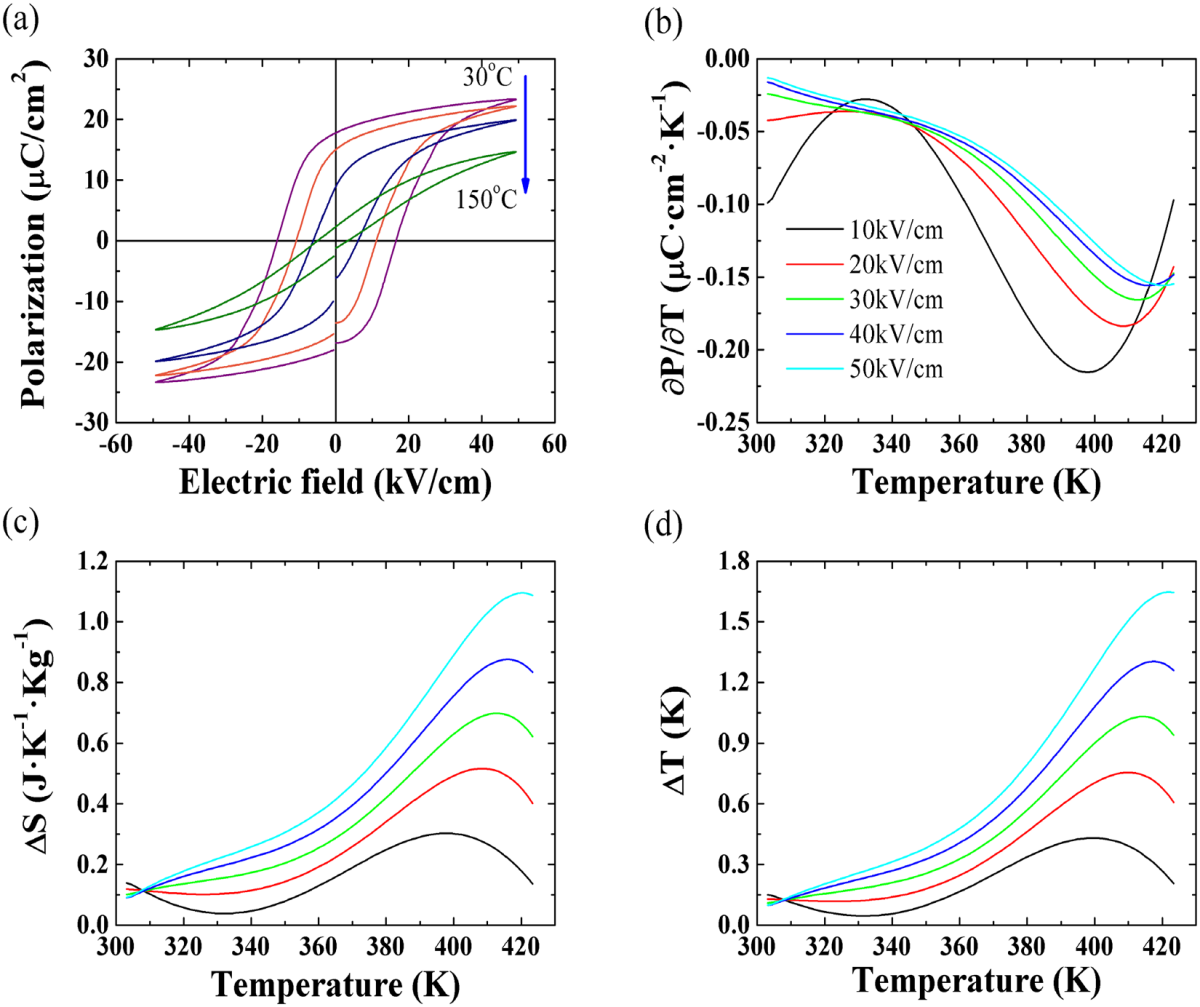
(**a**) Temperature dependent ferroelectric hysteresis loops. (**b**) ∂*P/*∂*T* curves of PLT20 samples. (**c**) The isothermal entropy change Δ*S* and (**d**) reversible adiabatic temperature change Δ*T* obtained from *P-E* data.

In order to calculate *ECE* of ceramics, ferroelectric properties of PLT20 (50 kV/cm, 30–150 °C) are showed in Fig. 8a. Values of saturated polarization decrease with increasing temperatures. According to the principle of *ECE*, when electric field increases from *E*_1_ to *E*_2_, the isothermal entropy change Δ*S* of an *ECE* material should be:

6 $${\rm{\Delta }}S=S({E}_{1},T)-S({E}_{2},T)$$

Therefore, initial conditions of *ECE* materials at *E*_1_ (in most cases, *E*_1_ = 0) will affect *ECE* directly. Assuming the Maxwell relation:

7 $${(\partial P/\partial T)}_{E}={(\partial S/\partial E)}_{T}$$

The corresponding isothermal entropy change Δ*S* and the reversible adiabatic temperature change Δ*T* are calculated by following relations^60,61^:

8 $${\rm{\Delta }}S=-1/\rho \cdot \int {(\partial P/\partial T)}_{E}dE$$

9 $${\rm{\Delta }}T=-T/C\rho \cdot \int {(\partial P/\partial T)}_{E}dE$$

where *ρ*, *C*, *E*_1_ and *E*_2_ are mass density, mass heat capacity, initial and final applied electric fields, respectively. Values of (∂*P/*∂*T*)_*E*_ (shown in Fig. 8b)can be obtained from the numerical differentiation of polarization*-*temperature data, which are extracted from upper branches of *P-E* loops (*E* > 0) measured at various temperatures.

Δ*S* and Δ*T* calculated at different electric fields are presented in Fig. 8c,d. Both Δ*S* and Δ*T* decrease firstly and then increase with increasing temperatures sharply (<30 kV/cm). On the other hand, Δ*S* and Δ*T* increase continuously with increasing temperatures especially for higher fields (>30 kV/cm). Temperatures of maximum Δ*T* (*T*_*ECmax*_) shift toward the higher temperatures with increasing electric fields. Although the ΔT value in this study is lower than PbZr_0.95_Ti_0.05_O_3_ film^19^, PMN-PT films^62^. But for bulk materials, our results show higher values of Δ*T* than Ba(Zr_0.2_Ti_0.8_)O_3_-(Ba_0.7_Ca_0.3_)TiO_3_ ceramics^13^, and Sr_0.75_Ba_0.25_Nb_2_O_6_ materials^63^. More comparison results^64–69^ are shown in Table 1.

**Table 1:** Comparison of *ECE* reported in this work with other bulk materials.

Material	*E*(kV/cm)	*T*(K)	*ΔT*_*max*_(K)	*§*_*max*_(K·cm·kV^−1^)	Method	Material
PLT(This work)	50	420	1.67	0.033	Indirect	Ceramic
0.75Bi_1/2_Na_1/2_TiO_3_-0.25SrTiO_3_^64^	40	375	≈0.4	0.010	Indirect	Ceramic
0.92Na_0.5_Bi_0.5_TiO_3_-0.8BaTiO_3_^65^	50	413	−0.33	−0.007	Indirect	Ceramic
BaHfTiO_3_^66^	50	338	1.35	0.027	Indirect	Ceramic
Ba_0.65_Sr_0.35_TiO_3_^67^	90	303	2.10	0.023	Indirect	Ceramic
0.9Pb(Mg_1/3_Nb_2/3_)O_3_-0.1PbTiO_3_^68^	57	380	≈1.25	≈0.0220	Indirect	Ceramic
Pb_0.85_La_0.1_(Zr_0.65_Ti_0.35_)O_3_^69^	200	—	3.1	0.0155	Indirect	Ceramic

*T*: Measured temperature.

In order to give a comparison criterion for electrocaloric refrigeration, refrigeration efficiency is given:

10 $$COP=|Q|/|W|=|{\rm{\Delta }}S\times T|/W$$

where *Q* and *W* are isothermal heat and corresponding electrical work per unit volume, and *W* is equal to ∫*EdP* ^70,71^. Our result reveals that value of *COP* for is about 27.4, which is much higher than previous reports of Pb_0.97_La_0.02_(Zr_0.75_Sn_0.18_Ti_0.07_)O_3_ thick film (*COP* = 18)^70^, PbZr_0.95_Ti_0.05_O_3_ film^19^, P(VDF-TrFE) film^72^, and PMN-PT (*COP* = 5.6) films^62^. Large values of *COP* suggest the high cooling efficiency, which implies PLT20 ceramics have potential applications in future solid-state refrigeration technologies.

In order to evaluate the quantitative effect of electric field Δ*E* on *ECE*, electrocaloric coefficient is given:

11 $${{\S }}_{max}={\rm{\Delta }}{T}_{max}/{\rm{\Delta }}{E}_{max}$$

where Δ*T*_*ma*x_ is the maximum temperature change and Δ*E*_*max*_ is the corresponding electric field change^70^. Clearly, maximum *§*_*max*_ achieved in this work is 0.033 K·cm·kV^−1^, which is higher than (Pb_0.97_La_0.02_)(Zr_0.67_Sn_0.38_Ti_0.05_)O_3_ thick films (0.030 K·cm·kV^−1^)^73^, BaZr_0.2_Ti_0.8_O_3_ ceramic^74^, and 0.94Bi_0.5_Na_0.5_TiO_3_-0.06KNbO_3_ ceramic^75^, 0.7Pb(Mg_1/3_Nb_2/3_)O_3_-0.3PbTiO_3_ (0.03 K·cm·kV^−1^)^15^, 0.9Pb(Mg_1/3_Nb_2/3_)O_3_-0.1PbTiO_3_ single crystal (0.025 K·cm·kV^−1^)^14^, 0.68Pb(Mg_1/3_Nb_2/3_)O_3_-0.32PbTiO_3_ thin films (0.022 K·cm·kV^−1^)^20^.

**Figure 9:**
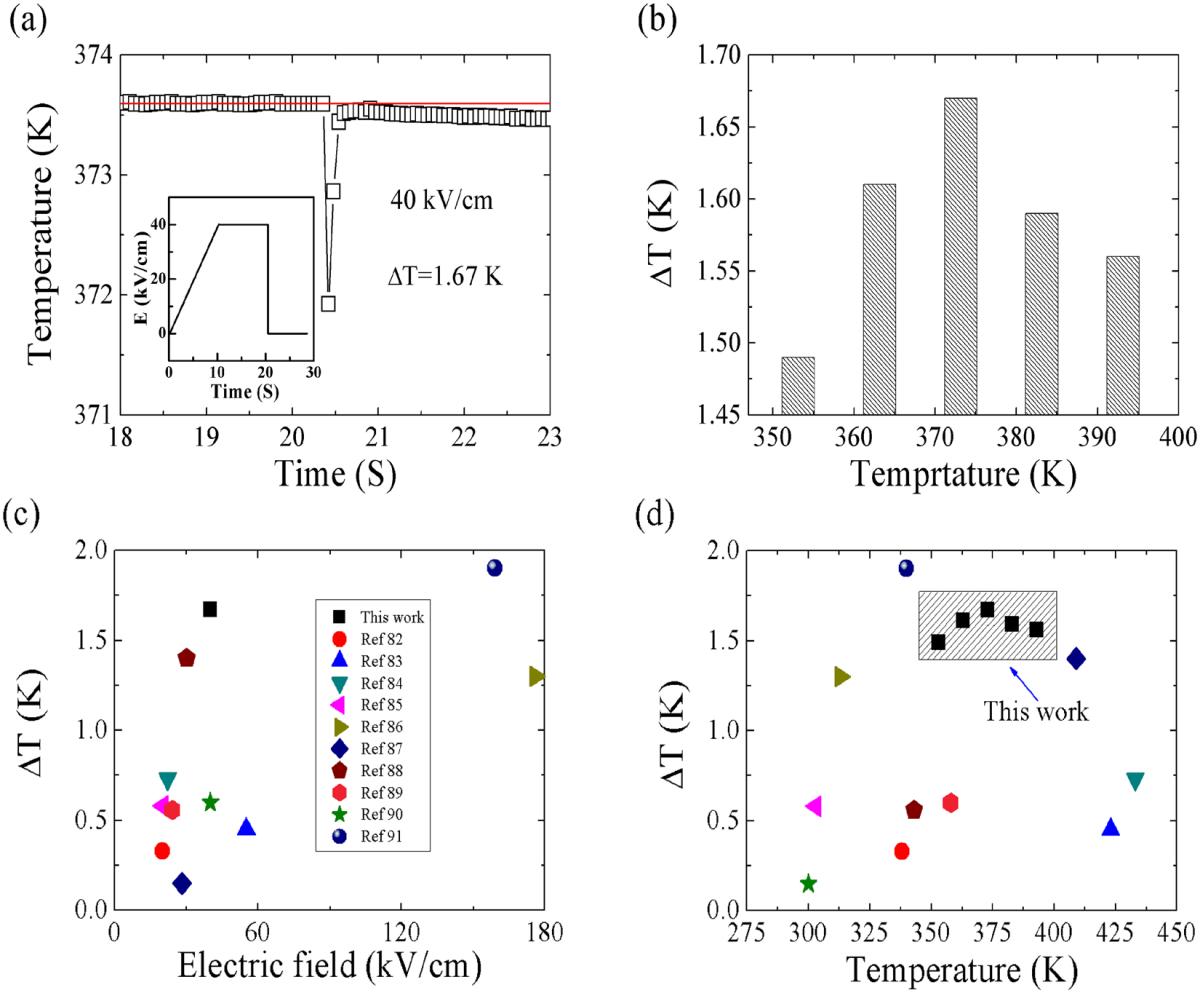
(**a**) Measured *ECE* data (open circles), insets show the schematic representation of the electric field pulse. (**b**) *ECE* data under various temperatures. The comparison of maximum reversible adiabatic temperature change Δ*T* reported under various measured electric fields (**c**) and temperatures (**d**) in this work with other bulk materials (direct measurement).

As PLT20 ceramic exhibits higher *ECE* in higher temperature region, so the directly measured Δ*T* (40 kV/cm) are analyzed from 353.15 to 393.15 K as shown in Fig. 9a,b. It is found that a subsequent removal of electric field produces a sudden decrease in temperature (1.67 K, shown in Fig. 9b) due to electrocaloric cooling. *§*_*max*_ calculated from direct measurements was about 0.050 K·cm·kV^−1^. Figure 9b shows the direct measurements under various temperatures, with increasing ambient temperatures, Δ*T* exhibits the maximum value at about 373.5 K. Although the temperature of maximum Δ*T* from theoretical calculation is higher than that of direct measurement, but they show the similar behaviours and are in good agreements. Compared to previous studies (direct measured *ECE*) on P(VDF-TrFE-CFE) film^76,77^, and P(VDF-TrFE) film^78^, the ΔT value in this study is smaller by one order of magnitude. But compared to that (directly measured *ECE*) of bulk materials, our measured results showed higher values than BaHfTiO_3_ ceramics^79^, PbMg_1/3_Nb_2/3_O_3_–30PbTiO_3_ single crystals^80^, PbZrO_3_ ceramics^81^. Figure 9c,d show the comparison of directly measured *ECE* reported here with some bulk materials^82–91^. For *ECE* researches on ceramics, Δ*T* (direct measurement) is usually very low, mostly below 1 K. Our research (maximum adiabatic temperature change) shows nearly 1.67 K, both electric field and temperatures dependent Δ*T* show high *ECE* values, and it may open more opportunities for practical application in refrigeration devices. The high ΔT value in this study indicates that PLT ceramics have potential applications in future solid-state refrigeration technologies.

Ferroelectrics that are characterized by the existence of an electric-field switchable polarization whose appearance is accompanied by structural phase transition have attracted increasing attention for the last 10 years especially in the field of *ECE* ^74^. Some strategies to enhance the *ECE* applications are possible, such as: maximizing the number of close-energy phases near a critical point in the temperature-composition phase diagram^74^, combining conventional and inverse caloric responses in a single refrigeration cycle^92,93^, introducing extra available degree of freedom like strain via mechanical stress^94^, and multicaloric effect driven by either single stimulus or multiple stimuli (applied/removed simultaneously or sequentially)^95^. Though promising, in bulk ferroelectrics, Δ*T* is usually less than a few kelvins, the obtained Δ*T* is still insufficient for practical application.

## Conclusions

In this work, PLT ceramics are prepared and studied. Relaxer phase transitions and high temperature relaxations are studied. Room temperature energy-storage density and energy-storage efficiency are about 0.31 J/cm^3^ and 91.2%, respectively. Temperatures and electric fields influenced energy-storage properties are analyzed. *ECE* is studied. High refrigeration efficiency (27.4) and large electrocaloric coefficient are achieved by theoretical calculation, maximum value of Δ*T* is about 1.67 K. Direct measurements of *ECE* shows that large Δ*T* (1.67 K) is obtained, such high value of directly measured Δ*T* is rare in previous reports.
